# Claw diseases are the dominant cause of lameness in dairy cows and a risk factor for mastitis

**DOI:** 10.17221/56/2024-VETMED

**Published:** 2025-02-24

**Authors:** Vladimir Hisira, Jana Zahumenska, Marian Kadasi, Robert Klein, Pavol Mudron, Frantisek Zigo

**Affiliations:** ^1^Clinic of Ruminants, University of Veterinary Medicine and Pharmacy, Košice, Slovak Republic; ^2^Department of Hygiene, Technology and Health Food Safety, University of Veterinary Medicine and Pharmacy, Košice, Slovak Republic; ^3^Department of Animal Nutrition and Husbandry, University of Veterinary Medicine and Pharmacy, Košice, Slovak Republic

**Keywords:** claw disease, cows, mastitis, mastitis score

## Abstract

Lameness and mastitis are two of the most economically important issues for the dairy industry. This study aimed to obtain a clearer analysis of the link between mastitis and lameness in dairy cows using the Mastitis Score and detecting the pathogens that predominate in dairy cows’ milk samples with claw diseases. During routine claw trimming, milk samples were collected from dairy cows in two Slovak dairy farms. Out of 558 cows presented for claw trimming, 144 cows (25.8%) exhibited lameness from claw diseases. The most prevalent disease in our study was digital dermatitis (43.1%), followed by toe necrosis (41.7%), and Rusterholz ulcer (15.3%). Udder inflammation in clinical form was diagnosed based on clinical examination of individual udder quarters, and sensory evaluation of milk from each quarter and in the subclinical form by the CMT. Mastitis was detected in 80 cows with claw diseases, clinical mastitis in 14 cows (17.5%), and subclinical mastitis in 66 cows (82.5%). On both farms, Mastitis Scores were significantly higher in dairy cows affected by claw diseases than in the non-lame ones. In mastitic cows affected by claw diseases, environmental pathogens dominated the infected milk samples. Our findings showed that cows with claw disease were more likely to have mastitis.

Mastitis is among the most common diseases of dairy cattle worldwide with an impact on the economy from reduced performance of dairy farms. Losses from clinical mastitis (CM) are reported to range from $179 to $518 on a “per cow-case” basis (Bar et al. 2008; Huijps et al. 2008; Hagnestam-Nielsen and Ostergaard 2009; Rollin et al. 2015), while losses from subclinical mastitis (SCM), defined as a somatic cell count (SCC) above 1 000 000/ml, averaged $360 (Tongel and Mihina 2004). The prevalence of mastitis has a wide variability concerning the farms’ geographical location, the technologies of dairy farming, and the types of housing (free stall, tie stall, dry lot, and pasture-based system). Krishnamoorthy et al. (2021) performed an extensive multi-year meta-analysis and calculated the prevalence of mastitis both worldwide and in India. When comparing the prevalence of mastitis on individual continents, they reported the highest subclinical mastitis (SM) prevalence was found in North America (46%), followed by Africa (44%), Asia (42%), Europe (37%), Oceania (36%), and Latin America (34%). The highest clinical mastitis (CM) prevalence was reported in Europe (29%) followed by other continents in the world, and the lowest was observed in Oceania (5%). In India, based on state-wise breakdown, the highest SCM prevalence was in West Bengal (75%) followed by Mizoram (65%), Chhattisgarh (63%), and Karnataka (58%). The highest CM prevalence in the world was in Sudan (77%), followed by the United Kingdom (74%), Sweden (59%), Kenya (54%), Uruguay (52%), Turkey (50%), Italy (47%), Finland (45%), Germany (43%), South Africa (37%) and the USA (35%) (Krishnamoorthy et al. 2021).

Mastitis is an inflammatory disease of the udder and represents a complex relationship between host, environment, and pathological agents. Therefore it can be considered as a multifactorial disease. Risk factors are divided into pathogen, environmental, and host factors, which are classified as individual and mammary gland factors (Radostits et al. 2007; Cervinkova et al. 2013; Ruegg and Petersson-Wolfe 2018).

The role of host factors involving a large group is one of the important aspects of mastitis prevalence. These host factors include phase of lactation, number of lactations, genetics, diseases associated with the peripartum period, other diseases such as lameness, breed, milk production, spontaneous milk leakage between two milkings, dry period, and cleanliness of the dairy cow. Mastitis affects animals at different stages of lactation. The higher incidence of mastitis is in the period immediately after parturition and in the early lactation phase (Nitz et al. 2021). Prevalence of mastitis is also influenced by the number of lactations, where with an increasing number of lactations there is an increase in incidence, and an increase in the number of somatic cells, which results in excessive culling of dairy cows (de Haas et al. 2002). This is probably an increased cellular response to intramammary infection or long-term damage to udder tissue in older animals. In younger animals, Dulin et al. (1988) suggested a more effective mechanism for host response to infection. In addition to the above-mentioned individual risk factors, genetics also plays an important role, where genomic selection has been carried out in the last decade to improve the performance and health indicators of the animal or herd. Traits for which genomic breeding values are established include production, body conformation, somatic cells, fertility, longevity, and genomic selection index (GSI), and also genomic breeding value (GEBV) for health traits (resistance to clinical mastitis, infectious and non-infectious diseases of the claws, and diseases of the limbs). In the case of genetically determined natural resistance based on genetic analysis of mastitis resistance in dairy cows, two possible methods of selection are known: selection of animals that contain alleles of certain genes in their genome with a marked effect on mastitis resistance, or animals that contain the joined effect of many genes, where each gene has a weaker effect on mastitis resistance (Detilleux 2002). The first group includes genes influencing resistance (genes coding for *TLR*, *CD18*, *BoLA DRB3*, *TNF*-α, i.e.) (Sender et al. 2003; Rupp et al. 2007; Wang et al. 2007). Another way is to select animals according to their genotypic indices [e.g. total immunocompetence indices, covering all aspects of immune system function (Kean et al. 1994)]. Nowadays, genomic selection in cattle breeding is part of everyday practice. In the Netherlands and Belgium, for example, about 72% of inseminations are carried out using genomically tested bulls. In Germany, the proportion of the bulls tested for genomics has remained around 80% in recent years, in the Nordic countries and France, the proportion is even higher. Likewise, in the USA, where large breeding farms take a very pragmatic approach, at least 80% of the insemination doses in dairy farms with Holstein cattle come from these bulls (Prymas 2021; Mamulova 2022).

In addition, host factors for mastitis also include diseases of the peripartum period [acidosis (Hu et al. 2022); ketosis (Suthar et al. 2013; Swartz et al. 2021)], but also others, such as lameness (Sato et al. 2008; Mudron 2016; Hisira et al. 2020).

## Scientific hypothesis

Claw diseases are recognised as the predominant cause of dairy cow lameness. It was hypothesised that lameness is one of the risk factors for mastitis, and so in this study, we compared the occurrence of mastitis in cows with and without lameness caused by claw diseases.

## MATERIAL AND METHODS

The study was conducted on two dairy cattle farms in the Košice district and Trebišov district, in the south of eastern Slovakia. The first herd consisted of 366 and the second of 192 dairy cows. All animals were housed in a free stall system. On both farms, the dairy cows were fed by TMR with maize and alfalfa silage as the main components.

All Holstein-Friesian dairy cows (558) in lactation were examined on both farms. The average milk yield on Farm 1 per year of a cow was 9 030 kg and on Farm 2 was 10 000 kg. The 2 herds in the study were similar in characteristics and management to those in southeastern Slovakia. On both farms, the cows were milked twice daily in a herringbone milking parlor (Agromont, Nitra, Slovakia) with twelve fixation boxes in two rows opposite each other. Prefoam+ (Hypred S.A., Dinard, France) was used for udder hygiene before milking and was applied as foam. After foaming, the teats were mechanically cleaned with dry paper wipes intended for cleaning the entire udder and for fore-stripping. The milking vacuum was set at 42 kPa with a pulsation ratio of 60 : 40 at a rate of 52 c/min. The milking was automatically stopped when the milk flow dropped to 0.2 l/min. The teats were disinfected by teat dipping with HM-VIR-Film (Agromont, Nitra, Slovakia) after the milking process. The milk was stored in refrigerated tanks at +5 °C and removed daily at around 11.30 a.m.

### Orthopaedic examination

During routine claw trimming and half-yearly orthopedic examination claw diseases were determined. The findings on the limbs and claws were diagnosed according to a new atlas with updated orthopedic terminology (Egger-Danner et al. 2015). Defects on the claws were professionally treated by veterinary practitioners and the obtained data were used for statistical analysis.

### Udder and milk samples examination

Clinical mastitis was detected by examination of the udder for visible signs of inflammation, such as reddish appearance, pain, or udder swelling, by detection of abnormal physical characteristics of the secretion (colour, consistency, smell) or the presence of pathological contents [flakes of casein, fibrin, pus, and blood; (Tancin 2013)] during the previously mentioned routine claw trimming. Visibly normal milk was examined by testing milk from each quarter with the modified CMT test (formation of flakes or gel, discolouration of the mixture). Before sampling for the modified CMT test, the first stream was milked out of the paddle and discarded. Then the milk sample was collected and examined from each quarter. A value of > 400 000 SCC/ml in the milk sample was considered a threshold for subclinical mastitis (Alhussien and Dang 2018).

### Bacteriological examination

Bacteriological examination focused primarily on the isolation of bacteria of the genus *Staphy-lococcus* spp., *Streptococcus* spp., *Enterococcus* spp., *E. coli*, *Bacillus* spp. The samples were inoculated onto Columbia agar plates supplemented with 5% sheep blood (Oxoid Ltd, Basingstoke, Hants, UK). These plates were then incubated at 37 °C and assessed after 24 hours. To more accurately determine the bacterial pathogens that cause mastitis, colonies from the blood agar plates were transferred to different selective culture media, including Edwards Medium, Staphylococcal Medium No. 110, and MacConkey Agar (Oxoid Ltd., Basingstoke, Hants, UK). The subcultures were then incubated for 24 h at 37 °C. Subsequently, tests for catalase activity, haemolysis, pigment formation, coagulase, and Gram staining were performed using the methods published by Malinowski et al. (2006). Species identification was performed using biochemical tests, including STAPHYtest 24, STREPTOtest 24, or ENTEROtest 24 (Erba Lachema, Brno, Czech Republic), and evaluated with the program TNW, v7.0 ProAuto (Erba Lachema, Brno, Czech Republic).

### Statistical analysis

Based on the results of the clinical examination of milk, we determined mastitis score for each cow by scoring each quarter and obtaining a total per cow using a new 6-step mastitis score system (healthy milk – 1, doubtful mastitis – 2, weak subclinical mastitis – 3, distinct subclinical mastitis – 4, strong subclinical mastitis – 5, clinical mastitis – 6) to evaluate the impact of claw disease on the incidence of mastitis in dairy cows. The final value of the Mastitis Score (MS) is the sum of points given to each udder quarter. If all udder quarters were healthy, the mastitis score was 4 per cow. In the case of clinical mastitis in all udder quarters, the mastitis score was 24 (Hisira et al. 2020).

The results were statistically compared between healthy cows and cows with claw diseases and among different locations of claw inflammation using the unpaired Student’s *t*-test and among individual claw diseases using the ANOVA test.

## RESULTS

During the claw trimming or orthopaedic examination, a total of 144 (25.8%) of the 558 cows examined were found to suffer from claw diseases. The most prevalent diseases in our study included digital dermatitis (43.1%), followed by toe necrosis (41.7%), and Rusterholz (sole) ulcer (15.3%, Table 1). Mastitis was found in 80 (55.6%) of the cows affected by claw diseases, with 14 (17.5%) of those exhibiting clinical mastitis and 66 with subclinical mastitis (82.5%). The prevalence of mastitis in animals was 50.0% or more for cows with any of the 3 claw diseases and the highest mastitis score (9.1) was found in cows with Rusterholz ulcers (Table 1). Of the total number of dairy cows from the first dairy farm (366), we detected claw diseases in 86 cows (23.5%, Table 2). The claw disease with the highest prevalence was toe necrosis (46.5%). This was followed by digital dermatitis (36.1%), and Rusterholz ulcer at 17.4%. Mastitis was detected in 46 (53.4%) cows with claw disease. This included 9 cows with clinical mastitis and 37 with subclinical mastitis (Table 2).

**Table 1:** The overall distribution of mastitis in 144 of 558 cows with claw diseases on 2 dairy farms in Slovakia

Claw diseases	Dairy cows with claw diseases (*n*/%)	Mastitis in cows with claw disease (*n*/%)	Clinical mastitis (number of cows)	Subclinical mastitis (number of cows)	Mastitis score (mean **±** SD)
Digital dermatitis	62 (43.1%)	31 (50.0%)	3	28	7.4 ± 3.39
Toe necrosis	60 (41.7%)	35 (58.3%)	6	29	8.7 ± 4.06
Rusterholz ulcer	22 (15.3%)	14 (63.6%)	5	9	9.1 ± 4,45
Totally	144 (25.8%)	80 (55.6%)	14	66	8.3 ± 3.71

SD = standard deviation

**Table 2:** Distribution of mastitis in individual claw diseases in 366 dairy cows on Farm 1

Claw diseases	Dairy cows with claw diseases (*n*/%)	Mastitis in cows with claw disease (*n*/%)	Clinical mastitis (number of cows)	Subclinical mastitis (number of cows)	Mastitis score (mean **±** SD)
Digital dermatitis	31 (36.1%)	14 (45.2%)	1	13	7.6 ± 3.96
Toe necrosis	40 (46.5%)	22 (55.0%)	4	18	8.5 ± 4.69
Rusterholz ulcer	15 (17.4%)	10 (66.6%)	4	6	9.7 ± 4.99
Totally	86 (23.5%)	46 (53.4%)	9	37	8.4 ± 4.50

SD = standard deviation

**Figure 1:**
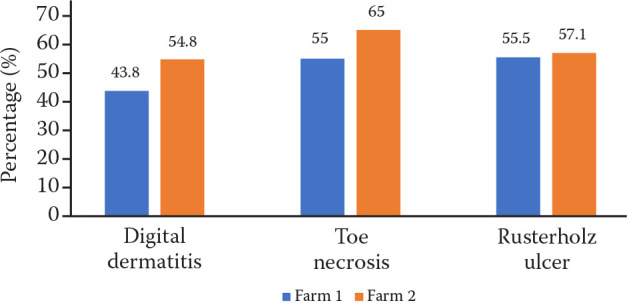
Distribution of mastitis among individual claw diseases

Among individual claw diseases, the highest proportion of animals with mastitis was for those affected by Rusterholz ulcer (55.5%). The prevalence of mastitis above 50% was also recorded in toe necrosis (Table 2, Figure 1).

For Farm 1, there no apparent differences in proportions of mastitis in the 3 types of claw lesions (Table 2) or mastitis scores in cows with inflammation of the corium as compared to cows with dermatitis (Table 3)*.*

**Table 3:** Mastitis scores in cows with inflammation in different areas of claws on Farm 1

Area of inflammation in claws	Dairy cows (*n*)	Mastitis score (mean ± SD)
Inflammation of the corium (toe necrosis, Rusterholz ulcers)	55	8.8 ± 4.76
Digital dermatitis	31	7.6 ± 3.96

SD = standard deviation

A total of 192 cows were examined on the 2^nd^ farm and claw diseases were found in 58 (30.2%) dairy cows. As shown in Table 4, digital dermatitis was the most common (53.4%), followed by toe necrosis (34.5%), and Rusterholz ulcer (12.1%). Mastitis was detected in 34 of the 58 cows with claw disease. This included 5 cows with clinical mastitis and 29 cows with subclinical mastitis (Table 4). The proportion of lame cows with mastitis was ≥ 54.8% or more for all 3 claw diseases. Out of a total of 58 claw diseases, inflammation of the corium occurred in 27 cases (46.6%), while digital dermatitis was reported in 31 cases (53.4%; Table 5) and no significant differences were found among them.

**Table 4:** Distribution of mastitis in individual claw diseases in 192 dairy cows on Farm 2

Claw diseases	Dairy cows with claw diseases (*n*/%)	Mastitis in cows with claw disease (*n*/%)	Clinical mastitis (number of cows)	Subclinical mastitis (number of cows)	Mastitis score (mean **±** SD)
Digital dermatitis	31 (53.4 %)	17 (54.8 %)	2	15	8.1 ± 3.26
Toe necrosis	20 (34.5 %)	13 (65.0 %)	2	11	7.9 ± 2.31
Rusterholz ulcer	7 (12.1 %)	4 (57.1 %)	1	3	9.0 ± 3.65
Totally	58 (30.2%)	34 (58.6 %)	5	29	8.2 ± 2.98

SD = standard deviation

**Table 5:** Mastitis score in cows suffering from different locations of inflammation in claws on Farm 2

Area of inflammation in claws	Dairy cows (*n*)	Mastitis score (mean ± SD)
Inflammation of the corium (toe necrosis, Rusterholz ulcers)	27	8.2 ± 2.69
Digital dermatitis	31	8.1 ± 3.26

SD = standard deviation

**Figure 2:**
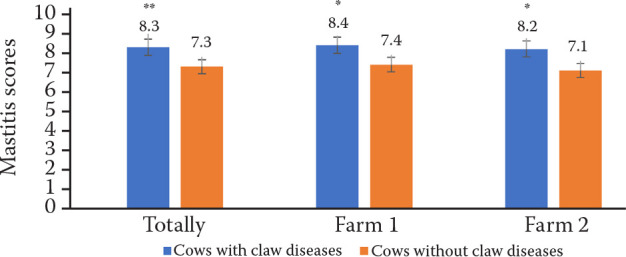
Mastitis scores (MS) in dairy cows with and without claw lesions *P < 0.05, **P < 0.01 (statistical significance)

For both Farms 1 and 2 individually, there were significant differences (*P* < 0.05) in mastitis scores when cows with claw disease were compared to cows without claw disease (Figure 2). For both farms collectively, the difference was highly significant (*P* < 0.01; Figure 2).

In both herds studied, a total of 80 cows were affected by claw disease and mastitis bacteria were detected in quarter milk samples of 59 cows (73.8%) (see Table 6). Of all intramammary pathogens isolated in the milk of dairy cows affected by claw diseases from the 2 herds, the highest incidence was recorded for environmental bacteria (78.0%, Table 6). Contagious pathogens were isolated from the remaining mastitis-positive samples. The breakdown between contagious vs environmental pathogens was similar for the 2 herds. However, as shown in Table 6, the individual bacteria differed somewhat between the 2 herds. *Streptococcus agalactiae* accounted for nearly all contagious pathogens in herd 1, while *S. aureus* accounted for all the contagious pathogens in herd 2. For environmental pathogens, CNS accounted for many of the isolates from both herds. As shown in Table 6, the remainder of the environmental pathogens were a variety of bacteria.

**Table 6:** Intramammary bacteria cultured from milk samples of cows with claw diseases

Intramammary pathogens	Farm 1	Farm 2	Together
Contagious pathogens	7	6	13 (22.0%)
*Streptococcus agalactiae*	6	0	6 (10.2%)
*Staphylococcus aureus*	0	6	6 (10.2%)
*Str. dysgalactiae*	1	0	1 (1.7%)
Environmental pathogens	27	19	46 (78.0%)
CNS	8	8	16 (27.1%)
*S. chromogenes*	2	1	3 (5.1%)
*S. warneri*	3	0	3 (5.1%)
*S. xylosus*	1	1	2 (3.4%)
*S. carnosus*	0	2	2 (3.4%)
*S. auricularis*	0	1	1 (1.7%)
*S. caprae*	0	1	1 (1.7%)
*S. epidermidis*	1	0	1 (1.7%)
*S. equorum*	0	1	1 (1.7%)
*S. felis*	1	0	1 (1.7%)
*S. scuri*	0	1	1 (1.7%)
*Enterococcus* spp.	8	6	14 (23.7%)
*Proteus* spp.	4	3	7 (11.9%)
*Aerococcus viridans*	3	0	3 (5.1%)
*S. intermedius*	0	1	1 (1.7%)
*Streptococcus* spp.	4	1	5 (8.5%)
*Str. porcinus*	2	0	2 (3.4%)
*Str. suis*	1	1	2 (3.4%)
*Str. salivarus*	1	0	1 (1.7%)
Totally	34	25	59

CNS = coagulase-negative staphylococci

The distribution of individual types of intramammary bacteria in dairy cows affected with various claw diseases was different, as indicated by the results of culturing (Tables 7 and 8). Although a relationship between claw disease as the most common cause of lameness and contagious intramammary pathogens is not assumed, in the case of digital dermatitis *S. aureus* dominated on Farm 2 (Table 8).

**Table 7:** Distribution of intramammary pathogens in positive milk samples of cows affected by claw diseases (Farm 1)

Claw disease	Intramammary pathogen
TN	16 (*Enterococcus* spp. 5, *Str. agalactiae* 3, *Proteus* spp. 2, *Str. dysgalactiae*, *Aerococcus viridans*, *S. chromogenes*, *S. epidermidis*, *S. xylosus*, *S. warneri*)
RU	7 (*Str. agalactiae* 2, *Aerococcus viridans*, *Enterococcus* spp., *Str. salivarus*, *Str. suis*, *Str. porcinus*)
DD	11 (*Enterococcus* spp. 2, *Proteus* spp. 2, *S. warneri* 2, *Str. agalactiae*, *Aerococcus viridans*, *S. chromogenes*, *S. felis*, *Str. porcinus*)

DD = digital dermatitis; RU = Rusterholz ulcer; TN = toe necrosis

**Table 8:** Distribution of intramammary pathogens in positive milk samples of cows affected by claw diseases (Farm 2)

Claw disease	Intramammary pathogen
DD	19 (*S. aureus* 6, *Enterococcus* spp. 3, *Proteus* spp. 3, *S. carnosus* 2, *S. chromogenes*, *S. xylosus*, *S. caprae*, *S. auricularis*, *Str. suis*)
TN	3 (*Enterococcus* spp., *S. scuri*, *S. equorum*)
RU	3 (*Enterococcus* spp. 2, *S. intermedius*)

DD = digital dermatitis; RU = Rusterholz ulcer; TN = toe necrosis

## DISCUSSION

Many risk factors, including lameness, influence the occurrence of mastitis. In a review of 53 studies from around the world, the prevalence of lameness ranged from 5.1% to 45%, and within the herd ranged from 0% to 88% (Thomsen et al. 2023). Lameness constitutes any foot or leg condition of infectious or non-infectious origin that negatively impacts cow mobility, posture, and gait (Archer et al. 2010; Garvey 2022) and affects the time of feeding and milk yield, body condition scores, reproductive and fertility performance, and increased culling (Huxley 2013). In Eastern Slovakia, the prevalence of mastitis reached 56% among cows with lameness (Mudron et al. 2017). However, in our study, the prevalence of lameness was lower.

In dairy cows, the main causes of lameness are claw lesions, which are either non-infectious traumatic (corkscrew claws, interdigital hyperplasia, asymmetric claws, scissors claws, and thin sole) or associated with metabolic disorders [laminitis, white line disease, sole (Rusterholz) ulcer, sole haemorrhage, double sole, horn fissure] and infectious, including digital dermatitis (DD), interdigital dermatitis (ID), heel erosion, interdigital phlegmon and swelling of coronet (Van Nuffel et al. 2015; Alvergnas et al. 2019). In Canadian herds digital dermatitis was the most common lesion among all housing types, present in 15% of cows. Sole ulcers and white line disease were detected in 6% and 4% of the cows (Solano et al. 2016). In a Swiss study from 2014–2021, an increasing prevalence was observed for DD (20.7%), white line disease (16.3%), and sole ulcer [4.6% (Becker et al. 2014; Jury et al. 2021)]. In the Netherlands, the prevalence of DD in several floor systems was 30% (Somers et al. 2003). As in previous research, DD dominated our study for claw disease (45.2%). Non-infectious claw disease was more prevalent (white line abscess 14.2 %, sole ulcer 9.7% and toe necrosis 8.9%).

Claw lesions causing lameness are generally perceived as painful for cows (Thomsen et al. 2012; Tschoner et al. 2021). Even in some cases, diseases of the claw area cause extreme pain with severe lameness and high locomotion scores (Whay et al. 1998). It was hypothesised that this pain forces dairy cows to spend most of their time lying down, which increases the risk of developing mastitis compared to healthy cows (Chapinal et al. 2010). Similarly, the duration of lying down plays an important role in the spread of mastitis. This lying interval is also extended in case of diseases of the claws affecting the joint. A very long period of lying in dairy cows results in high exposure to pathogenic microorganisms in the environment and the formation of mastitis (Singh et al. 1993). Consistent with this claim, our study demonstrated the dominance of environmental bacteria in dairy cows affected by claw diseases. However, a recent study detected environmental contamination by contagious bacteria (*S.* *aureus*), both in the housing and in the milking parlour (Vargova et al. 2023).

Environmental and body hygiene in dairy cows plays a significant role in udder exposure to intramammary pathogens and is a major source of environmental mastitis in dairy cows (Bartlett et al. 1992; Zigo et al. 2020). A Canadian study detected a close relationship between poor hygiene in dairy cows and an increased number of somatic cells. In the study looking at the influence of the length of time from the end of milking to the start of lying down, it was found that dairy cows that lie down soon after milking have an increased risk of “acquiring” an intramammary infection caused primarily by environmental pathogens, which is probably due to non-closure of teat ducts (DeVries et al. 2011). Also, poor hygiene on stands increases the predisposition of claw and mammary gland infections (Cook and Nordlund 2003).

A positive relationship was observed between poor foot health and a high incidence of clinical mastitis (Arvidson 2000). A similar relationship between poorly trimmed claws, lameness, milk yield, and lying and rising behaviour was also reported by Rajala-Schultz and Grohn (1999). In a large-scale study of dairy farm conditions in the USA, the prevalence of intramammary pathogens was found to be significantly correlated with udder hygiene scores (Schreiner and Ruegg 2003).

According to previous data, claw diseases are predicted as the main cause of increased teat and mammary gland contamination, contributing to an increased risk of mammary gland infections in dairy herds.

Our findings were consistent with those of several studies that confirmed the effect of lameness on mastitis incidence in cattle farms (Peeler et al. 1994). Also, Sato et al. (2008), in a study on Danish farms, found that claw diseases significantly influenced the incidence of clinical mastitis in herds. Previous studies emphasise that the hygiene of the environment and providing claw trimming to dairy cows are important components of a program to control mastitis. A hygienic environment reduces the occurrence of pathogens, and the main effect of foot therapy is that it decreases the lying time of cows due to soreness in the limbs and udder contamination. Both these practices significantly decrease bacterial spreading, transmission, and subsequent intramammary infection.

In a study conducted in India, it was concluded that lame cows had significantly higher SCC milk and poor quarterly health compared to healthy cows (Singh et al. 2018). According to Indian research, average values of somatic cell count (SCC) among lame animals with and without subclinical mastitis (SCM) showed significant differences (Dogra et al. 2020). Zhang et al. (2015) also reported increased SCC levels in lame cows and a positive correlation between lactate, interleukin 6 (IL-6), tumour necrosis factor-α (TNF-α), and serum amyloid (SAA), further suggesting that mammary gland infection before dry-off could contribute to the development of diseases post-partum. In monitoring the impact of individual claw diseases on the prevalence of mastitis in dairy herds an association was found between clinical mastitis in the first 30 days in milk (DIM) and the presence of sole ulcer in early lactation [after 30 DIM (Watson et al. 2022)]. No relationship between individual hoof diseases has been statistically confirmed by the mastitis score in our work. Nevertheless, in both farms, in cases of sole ulcer and white line abscess, mastitis occurred in at least half of the affected dairy cows with these diseases.

Contrarily in 2012, Olechnowicz and Jaskowski (2012) evaluated the effect of lameness on somatic cell count (SCC) but found no significant increase in SCC in lame cows compared to healthy cows. Also, Heuer et al. (1999) found no relationship between mastitis and lameness, pointing out the low percentage of mastitis incidence in cows with lameness. Hultgren et al. (2004), who did not confirm a close relationship between lameness and mastitis in their study, also came to the same results.

Lameness and mastitis cause significant economic losses to the dairy industry worldwide. Mastitis is caused by many pathogens, and their prevalence is influenced by several risk factors. According to recent studies, lameness is also one of these risk factors, because lame dairy cows affected by various diseases of the limbs (claws, joints, or bones) are thought to spend much more time lying down. When lying down, dairy cows are exposed to environmental bacterial contamination from soiled bedding, which increases the risk of mastitis in lame dairy cows, whether in clinical or subclinical form. Therefore, this study was focused on monitoring the effect of lameness on the incidence of mastitis on two farms where claw diseases were the predominant cause of lameness. A statistical analysis of mastitis scores showed that when all claw diseases on both farms were considered, there was an increased incidence of mastitis in lame dairy cows. However, when analysed separately, no significant differences were found between individual claw diseases and mastitis prevalence. This suggests that lameness is a significant risk factor for mastitis in dairy farms and contributes to economic losses caused by a decline in the quantity and quality of dairy production.
